# Supplementary material for: Identification of photoperiod-induced specific miRNAs in the adrenal glands of Sunite sheep (Ovis aries)
Source: Front Vet Sci. 2022 Jul 22;9:888207. doi: 10.3389/fvets.2022.888207 (PMC9354845; doi:10.3389/fvets.2022.888207)
Supplement: Supplementary file 1 [file Data_Sheet_1.ZIP › Supplementary file/miRDeep2 file.zip!/miRDeep2 file/Know_Structure/SPLP42A1/oar-mir-1193.pdf]

miRBase precursor : oar-mir-1193
Total read count : 3
oar-miR-1193-5p read count : 3
oar-miR-1193-3p read count : 0
remaining reads : 0

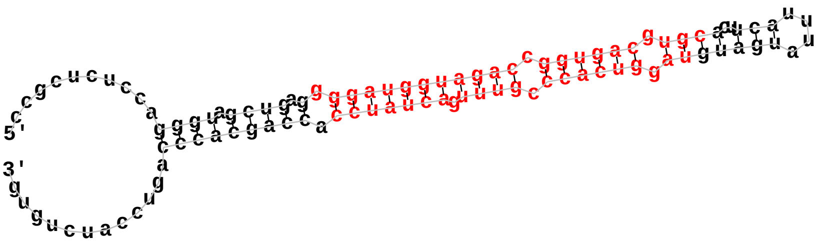

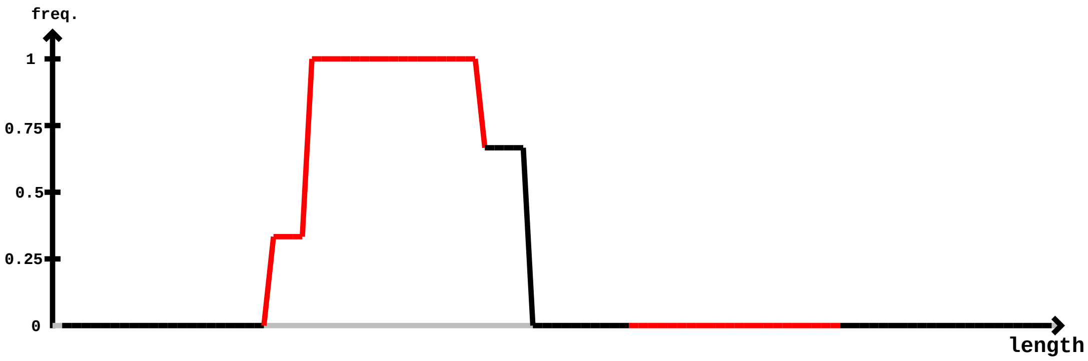


```
                              oar-miR-1193-5p
                                                              oar-miR-1193-3p
5'- ccgcucuccaggguagcugaggggaugguagaccggugacgugcacuucauuuaugauguaggucacccguuugacuaucaccagcacccaguccaucugug   -3'    exp
    ..........((((.((((.(.(((((((((((.((((((.((((..((((....))))))).)))))).)))).))))))).))))))))))............   reads    mm    sample
    .....................gggaugguagaccggugacgug.............................................................   1        0     seq
    .........................ugguagaccggugacgugcacuu........................................................   2        0     seq
```
